# Health Risks for Consumers of Forest Ground Cover Produce Contaminated with Heavy Metals

**DOI:** 10.3390/toxics12020101

**Published:** 2024-01-24

**Authors:** Magdalena Niezgoda, Grzegorz Dziubanek, Danuta Rogala, Anna Niesler

**Affiliations:** 1 School of Public Health in Bytom, Medical University of Silesia in Katowice (Poland), ul. Piekarska 18, 42-902 Bytom, Poland; 2 Department of Environmental Health Risk Factors, School of Public Health in Bytom, Medical University of Silesia in Katowice (Poland), ul. Piekarska 18, 42-902 Bytom, Poland; gdziubanek@sum.edu.pl; 3 Department of Environmental Health, School of Public Health in Bytom, Medical University of Silesia in Katowice (Poland), ul. Piekarska 18, 42-902 Bytom, Poland; anna.niesler@sum.edu.pl

**Keywords:** edible mushrooms, berries, heavy metals, oral exposure, health risks

## Abstract

Background: The activity of heavy metal (HM) mining and processing industries causes soils contamination with HM. The metals could be transferred from contaminated soils to edible plants and fungi. This study aimed to assess the content of Cd, Pb, Hg, As, and Ni in berries and edible mushrooms collected in the forests located near Miasteczko Slaskie zinc smelter and in the Lubliniec region, in the context of consumers’ health risk. Methods: The ET-AAS method was used to determine the content of Cd, Pb, Ni, and As. Mercury concentration was determined using the CV-AFS method. Results: The studies showed high levels of Cd and Pb in the examined products. A statistically significant impact of the distance from the smelter on the Cd concentration in the berries was observed. Total non-cancer health risk from the combined exposure of adults to all HM in mushrooms and berries was significant when consuming the most heavily contaminated produce. The risk to children was significant, even when consuming products with moderate levels of the metals. Ingestion of Cd by children with mushrooms was related to a high cancer risk. The uncertainty of the results was determined. Conclusions: It is recommended to take action to increase awareness among residents of the areas adjacent to the forests regarding the existing health risk and to take possible measures to reduce exposure to HM.

## 1. Introduction 

Exposure to heavy metals is considered one of the most pressing environmental issues in both industrialized and developing countries [1]. For the general population, the most significant route of exposure to metals is through food. Edible plants, mainly vegetables and cereal products, account for the highest intake of metals such as cadmium, lead, and zinc [2]. At the same time, fish and seafood are the primary sources of mercury exposure [3]. Edible mushrooms and forest berries are also potential sources of exposure to toxic metals for the human body. The hyphae of edible mushrooms demonstrate high capabilities for accumulating heavy metals, especially when they grow in soils heavily contaminated with metals [4]. Metal accumulation also occurs, although to a lesser extent, in fruits of plants that make up the forest ground cover. The research for this study was conducted in the Silesian Voivodeship, located in southern Poland, within the influence area of the zinc smelter ‘Miasteczko Śląskie’ S.A., where the prevailing winds are west and south-west. The average wind speed in this area is V = 2.92 m/s, with the strongest winds from the south-west (V = 3.92 m/s) and west. Poland is situated in Central Europe, in a temperate climate zone, with an average annual temperature of 9.5 °C and an average annual rainfall of 534.4 mm [5]. The Silesian Voivodeship is the most heavily polluted region with heavy metals in the country, housing the extensive forest complex known as the ‘Lasy Lublinieckie’ (Lubliniec Forests). The complex is part of the forest protection belt established in 1968 within the Upper Silesian Industrial Region, intended to neutralize the harmful impact of industry [6]. Overall, the forest area in Poland at the end of 2021 was 9264.7 thousand hectares, constituting 29.6% of the country’s total land area [7]. The Lubliniec Forests complex covers an area of almost 100 thousand hectares, of which 82% are pine forests. The forest substrate contains small amounts of mineral resources such as iron ore. There are three types of soils in the complex: swamp soils, podzols, and rusty soils, and typical brown soils. The Lubliniec Forests, together with the Rybnik and Pszczyna forests, belong to the so-called Forest Protection Belt of the Upper Silesian Industrial Region and are considered to be the so-called ‘Lungs of Silesia’ [8]. The studied area has been associated with mining and processing of non-ferrous metal ores for centuries. Extraction of non-ferrous metal ores ceased in the 1920s [9]. Currently, the only active industrial plant dealing with the processing of non-ferrous metals is the zinc smelter ‘Miasteczko Śląskie’ S.A. The feedstock contains a mixture of zinc-lead concentrates (zinc blende and galena), which is used in obtaining zinc and lead by the Imperial Smelting Process (ISP). In addition to useful components such as zinc and lead sulfides, the feedstock also contains ores such as pyrite, rich in arsenic, thallium, cobalt, nickel, copper, barium, magnesium, and strontium. Consequently, the dust emitted into the environment contains significant amounts of toxic elements [10]. As a result, environmental pollution, particularly of soils with heavy metals, occurs [11,12].

Residents of the Upper Silesian conurbation still engage in gathering forest ground cover produce, such as mushrooms and berries, as a way to spend their leisure time. The health safety of produce obtained from forest complexes located within the influence of industrial plants is much lower than in the case of items purchased in stores, which are subject to quality monitoring and procedures to ensure the highest product quality [13,14]. In recent years, there has been an increase in the consumption of mushrooms in many European countries. Studies conducted in various countries have shown that the consumption of mushrooms is declared by more than 80% of consumers [15]. Mushroom consumption in Poland is widespread, mainly due to the taste and aroma qualities. Mushrooms serve as both a staple and an addition to many dishes in Polish cuisine. It is not recommended for children under the age of twelve to consume wild mushrooms, although in some cases they are eaten also by children. Mushrooms are consumed practically all year round, because, apart from fresh mushrooms available during the growing season, mushrooms are also available in dried, frozen, or pickled forms. Biologically active compounds in edible mushrooms exhibit various health-promoting properties, such as anticancer, anti-inflammatory, antioxidant, and immune-stimulating effects [16]. In turn, bilberries (*Vaccinium myrtillus*), commonly called ‘blueberries’ in Poland, are consumed as ingredients in desserts and main dishes. Like mushrooms, bilberries are available to consumers throughout the year, fresh during the growing season, and in frozen or processed forms off-season. Berries are rich in antioxidants, containing high levels of anthocyanins, which exhibit strong antioxidant properties. The higher the content of anthocyanins, the higher the antioxidant activity [17].

Exposure to the metals considered in the study poses a significant threat to human health. Strong pro-oxidative properties characterize heavy metals; even minimal exposure of the general population to toxic elements can lead to tissue and organ damage [18]. Chronic lead exposure results in Central Nervous System (CNS) disorders. Lead is particularly dangerous for children, as it contributes to a decrease in Intelligence Quotient (IQ), weakens concentration, memory, and learning abilities, leads to school underachievement, apathy, irritability, drowsiness, and also causes speech and hearing impairments, and other effects [19,20]. According to the International Agency for Research on Cancer (IARC), cadmium is classified as a human carcinogen. Exposure to cadmium may increase the risk of developing various malignant tumors, including lung cancer, prostate cancer, or breast cancer [19,21]. Mercury exhibits nephrotoxic properties and disrupts the functioning of the nervous system, leading to symptoms such as paralysis, motor coordination disturbances, balance disorders, insomnia, memory loss, and seizures [22]. The symptoms of arsenic exposure primarily involve skin changes, such as hyperpigmentation and hyperkeratosis. Skin keratoses can transform into malignant skin tumors [19]. The most commonly observed effect of human exposure to nickel is allergies manifested as itchy eczema, for example, on the hands or forearms [23,24]. Additionally, nickel is a carcinogen [25]. In the study, it was assumed that the long-term emission of heavy metals from the zinc smelter ‘Miasteczko Śląskie’ S.A. contributed to severe environmental contamination with toxic metals, especially in the soils within the facility’s impact area. It was also assumed that the accumulation of heavy metals in the soils resulted in the contamination of forest ground cover produce, namely edible mushrooms and berries, originating from local forest ecosystems. The study aimed to assess the content of selected toxic metals, such as cadmium, lead, mercury, arsenic, and nickel, in edible berries and mushrooms collected in the forests located in Miasteczko Śląskie and Lubliniec region, focusing on evaluating consumers’ exposure and health risks. 

## 2. Material and Methods 

### 2.1. Sample Collection

**Figure 1:**
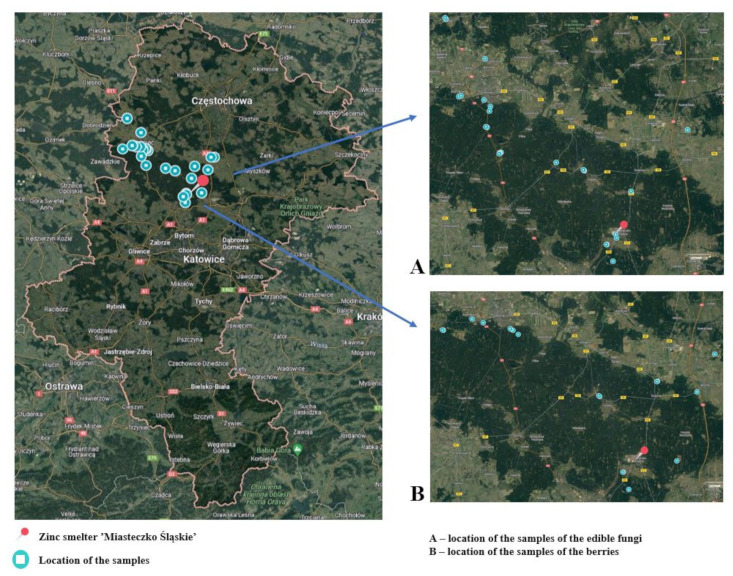
Location of the sample collection points of the edible mushrooms and the blueberries. Source: Own study based on Google Maps.

The research material consisted of samples of forest ground cover produce collected from areas near the Zinc Smelter ‘Miasteczko Śląskie’ S.A. Sampling sites were randomly selected at different distances from the smelter. Samples of bilberries, the fruits of *Vaccinium myrtillus* (N = 26), as well as edible mushrooms (N = 26), were gathered for the study. The samples collected consisted of about 0.5 kg of fresh bilberries with an average moisture content of 84% and 3–4 fresh mushrooms with a moisture content of 85–95%. Among the collected mushrooms were species such as *Xerocomus subtomentosus* (suede bolete), *Boletus badius* (bay bolete), *Leccinum aurantiacum* (red-capped scaber stalk), *Leccinum scabrum* (birch bolete), *Boletus edulis* (porcini), and *Suillus luteus* (slippery jack). Each time, the sampling locations were recorded in terms of latitude and longitude coordinates. Based on geolocation data, the distance of the sampling sites for edible mushrooms and berries from the zinc smelter ‘Miasteczko Śląskie’ S.A. was determined (Figure 1). The samples have been divided into two groups based on distance from the pollution center: zone S1 and zone S2. Group S1 samples were collected from locations within a 15 km radius of the zinc smelter. Group S2 samples were collected from locations situated more than 15 km away (S2) from the zinc smelter. A detailed description of the sampling sites is presented in Table S1. 

### 2.2. Preparation of the Study Material

The examined samples of fresh berries and edible mushrooms were homogenized into a smooth, uniform mass using the T18 Digital Ultra-Turrax homogenizer from IKA (Staufen, Germany) with a stainless-steel tip and titanium blade. From each sample, a weighed amount of 2 g (±0.06 g) was prepared using a precision laboratory scale model PS 750/X from RADWAG (Radom, Poland), equipped with an anti-draft chamber. The weighed amounts were transferred to Teflon vessels, and 8 mL of 65% ultrapure nitric acid (Merck (Rahway, NJ, USA)) and 1 mL of 30% hydrogen peroxide (Stanlab (Lagos, Nigeria)) were added. Subsequently, the vessels were sealed and placed in a microwave digester (Ertec’s Magnum II (Nuenen,The Netherlands)). The samples underwent a 4-stage mineralization process:

- Stage I: Mineralization time 10 min/60% power, pressure 17–20 bar, temperature: 200–220 °C;
- Stage II: Mineralization time 10 min/80% power, pressure 25–28 bar, temperature: 200–220 °C;
- Stage III: Mineralization time 10 min/100% power, pressure 32–35 bar, temperature: 200–220 °C;
- Stage IV: Cooling the sample for 15 min.

The mineralized samples were transferred to disposable measuring flasks with a capacity of 50 mL and diluted with ultrapure water to a predetermined volume. 

### 2.3. Sample Analysis

In the mineralized samples, the concentrations of elements such as Cd, Pb, As, and Ni were determined using Electrothermal Atomic Absorption Spectrometry (ET-AAS) with a graphite furnace, utilizing the SavantAA Sigma atomic absorption spectrometer from GBC. Meanwhile, the mercury concentration was determined using Cold Vapor Atomic Fluorescence Spectroscopy (CV-AFS) with the Millennium Merlin 10.025 total mercury analyzer from PSAnalytical. 

### 2.4. Quality Control and Quality Assurance

In order to create the calibration curve, the Certificate of Reference Material Cadmium and Lead Matrix: 1000 µg/mL in 2% Nitric Acid (AccuStandard, New Haven, CT, USA), Arsenic and Nickel Matrix: 1000 µg/mL in 5% Nitric Acid (Agilent Technologies, Santa Clara, CA, USA), and Mercury Matrix: 999 ± 5 µg/mL in 10% Nitric Acid (SCP Science) were used. To confirm the correctness of analytical measurements, the reference material (RM) for tomato paste was used (Certificate of Reference Materials TYG076RM). The limit of quantification (LOQ) of the method was 0.008 mg Cd/kg, 0.080 mg Pb/kg, 0.0005 mg Hg/kg, 0.43 mg As/kg, and 0.83 mg Ni/kg. The results of metals content below LOQ were not included in the health risk calculations.

## 3. Health Risk Assessment

The assessment of exposure and health risks to consumers of the examined forest ground cover produce from the impact area of the zinc smelter ‘Miasteczko Śląskie’ S.A. was conducted using the method developed by the United States Environmental Protection Agency (US EPA). First, the metal dose ingested through food consumption was calculated for adults and children, considering the following exposure scenarios:

I. Consumption of mushrooms with the lowest metal content;
II. Consumption of mushrooms with moderate metal content;
III. Consumption of mushrooms with the highest metal content;

Ia. Consumption of berries with the lowest metal content;
IIa. Consumption of berries with moderate metal content;
IIIa. Consumption of berries with the highest metal content.

Additionally, exposure levels and health risks for consumers of mushrooms and berries collected near Miasteczko Śląskie and in the Lubliniec forests were compared, dividing the study area into two zones. The first zone comprised the area within a 15 km radius (S1) of the Miasteczko Śląskie zinc smelter, while the second zone included the area beyond 15 km (S2) from the zinc smelter. The dose ingested by consumers was estimated using the following formula.

(1) $$\mathrm{Ingested}\;\mathrm{Dose}=\frac{C\;[\mathrm{mg}/\mathrm{kg}]\;\times \;V\;[\mathrm{kg}/\mathrm{day}]}{\mathrm{BW}\;[\mathrm{kg}]\;},$$

C—the concentration of the metal in mushrooms or berries (mg/kg);

V—the volume of mushrooms or berries consumed per day([kg/day]);

BW—the body weight of the exposed individual (kg).

Based on the research conducted by Kalac and Svoboda in 2000 [26], it was assumed that the average weight of mushrooms consumed by adult consumers is 0.0273 kg/day. Additionally, according to the study conducted by Kantar Polska, it was established that the average daily weight of consumed berries is 0.0027 kg/day [27]. In the case of children, the daily consumption of mushrooms and berries was assumed to be half of that of adults: 0.0136 kg/day and 0.0014 kg/day, respectively. The exposure assessment assumed that the average body weight of adults was 70 kg and that of children was 15 kg. Non-cancer health risks were assessed by calculating the Hazard Quotient (HQ), comparing the estimated exposure dose with the Reference Dose (RfD) or Benchmark Dose Lower Confidence Limit (BMDL) determined for specific elements under oral exposure conditions, according to the formula:

(2) $$\mathrm{Hazard}\;\mathrm{Quotient}\left(\mathrm{HQ}\right)=\frac{\mathrm{daily}\;\mathrm{dose}\;\mathrm{taken}}{\mathrm{RfD}\vee \mathrm{BMDL}}$$

The threshold dose values are presented in Table 1. If the calculation result equals or exceeds unity (HQ ≥ 1), it is considered that exposure to the specific element poses a significant health hazard. However, when the hazard quotient value is less than unity (HQ < 1), the exposure is considered insignificant in terms of non-cancer health risk. The uncertainty analysis of the modelled Ingested Dose and Hazard Quotient values was conducted by dividing the standard deviation by the square root of the number of measurements. Cancer risk (CR) was calculated for cadmium by multiplying the ingested dose with a cancer slope factor (SF), which is defined as a plausible upper-bound estimate of the probability of a response per unit intake of the chemical over a lifetime. The value of SF for Cd is 380 µg/kg/day [28].

Cancer Risk (CR) = Ingested dose × Slope Factor (SF)(3)

Cancer risks in the range of 1.0 × 10^−6^ to 1.0 × 10^−4^ are acceptable, higher than 1.0 × 10^−4^ is considered unacceptable, but greater than 1.0 ×10^−3^ is a risk requiring intervention. The statistical analysis of the results was conducted using Statistica software (StatSoft Polska Sp. z o.o., Kraków, Poland) version 13.3. The normality of the distribution of the variables was assessed using the Shapiro–Wilk test, and the Mann–Whitney U test was used in further statistical analyses. A significance level of *p* < 0.05 was adopted for all tests.

## 4. Results

### 4.1. Sample Contamination by Heavy Metals

The studies revealed that the average cadmium content in the analyzed mushrooms was 0.98 mg/kg, nearly twice as high as the permissible concentration limit [34]. Among the 26 tested fungi, elevated concentrations were detected in as many as 16 samples. The most heavily contaminated were boletus samples (3.35 and 3.01 mg Cd/kg f.m.) (Table 2 and Table S2). The cadmium content in mushrooms collected from locations within a 15 km radius (S1) of the ‘Miasteczko Śląskie’ S.A. zinc smelter averaged 1.24 mg/kg f.m. Among the ten samples, as many as six contained cadmium concentrations exceeding the highest permissible limit. In the case of mushrooms collected from locations situated more than 15 km away (S2) from the ‘Miasteczko Śląskie’ Zinc Smelter, the average cadmium content was 0.82 mg/kg fresh weight. Among the sixteen analyzed samples, as many as ten had concentrations exceeding the maximum permissible level.

The average lead content in the examined mushrooms was 0.60 mg/kg, whereas the highest permissible concentration reached 0.80 mg/kg f.m. [34]. In ten samples, the metal content did not exceed the limit of quantification (LOQ < 0.08 mg/kg f.m.). Elevated concentrations were found in four samples. The most heavily contaminated samples were the Suillus luteus: 1.75 and 2.05 mg Pb/kg f.m. (Table 2 and Table S2). The average lead concentration in mushrooms collected from locations within a 15 km radius (S1) of the zinc smelter was 0.68 mg/kg fresh weight. Only in one sample was the lead content lower than the limit of quantification (LOQ). Lead concentrations exceeding the maximum allowable limit were found in three mushroom samples. The average lead concentration in mushrooms from zone S2 (more than 15 km) was 0.51 mg/kg f.m. Only one sample had a lead content exceeding the maximum permissible level, while in nine samples the metal concentration remained below the limit of quantification (LOQ). 

The mercury concentration in fungi averaged 0.0274 mg/kg f.m. In ten of them, the mercury content did not exceed the limit of quantification (LOQ < 0.0005 mg/kg f.m.). None of the examined mushrooms exceeded the maximum permissible mercury concentration of 0.50 mg/kg f.m. [34]. The most contaminated sample originated from the Kokotek locality and was the porcini, containing 0.124 mg/kg f.m. of mercury (Table 2 and Table S2). In mushrooms from zone S1 (within a 15 km) the average mercury content reached 0.014 mg/kg f.m. In four of the examined mushrooms, the mercury concentration did not exceed the limit of quantification (LOQ) (Table 2 and Table S2). In samples of mushrooms collected from zone S2 (more than 15 km), the average concentration reached 0.036 mg/kg f.m. Mercury content exceeded the limit of quantification (LOQ) in ten samples, while in the remaining six it was lower than 0.0005 mg/kg f.m. (Table 2 and Table S2).

None of the examined mushrooms showed values exceeding the limit of quantification (LOQ) for arsenic (<0.43 mg/kg f.m.) and nickel (<0.83 mg/kg f.m.) (Table 2 and Table S2). 

**Figure 2:**
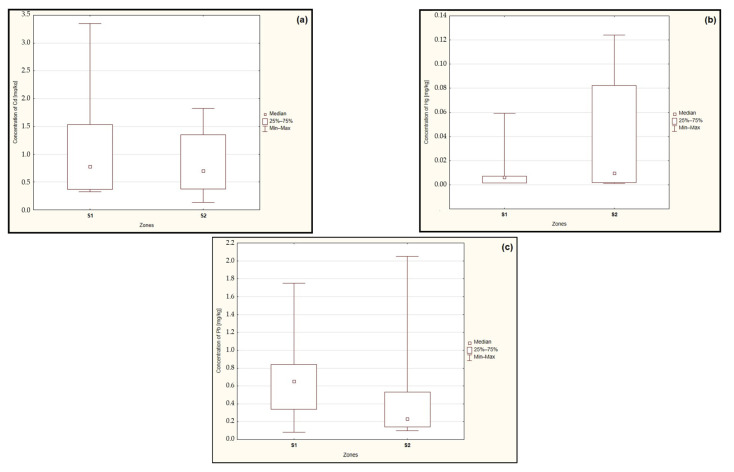
Median concentration of cadmium (**a**), mercury (**b**), and lead (**c**) in the edible fungi from zones located up to 15 km (S1) and above 15 km (S2) from the heavy metal emitter. Source: Own study.

The results of the statistical analysis conducted using the Mann–Whitney U test did not demonstrate statistically significant differences in the median cadmium (*p* = 0.493), mercury (*p* = 0.415), and lead (*p* = 0.314) concentrations in the examined mushrooms based on the distance of the sampling location from the ‘Miasteczko Śląskie’ zinc smelter (Figure 2a–c). 

Research on the content of cadmium in blueberries collected from locations in the area of Miasteczko Śląskie and Lubliniec Forests showed that the average concentration of this metal was 0.05 mg/kg f.m., surpassing the maximum permissible level of 0.03 mg/kg f.m. [34]. In only five out of 26 samples, the cadmium content was below the limit of quantification (LOQ). The most heavily contaminated blueberries were those collected from the forest near Bibiela and in Miasteczko Śląskie, where the cadmium content was: 0.20 mg/kg f.m., 0.19 mg/kg f.m., 0.18 mg/kg f.m., and 0.12 mg/kg f.m., respectively. The cadmium concentration in these fruits was 4.0 to 6.7 times higher than the maximum permissible level (Table 2 and Table S3). In fifteen samples of blueberries collected from locations within a 15 km radius (S1) of the ‘Miasteczko Śląskie’ zinc smelter, the average cadmium content was 0.71 mg/kg f.m., which was 2.4 times higher than the maximum permissible concentration. Excessive cadmium content was observed in as many as eleven out of the fifteen analyzed samples. The cadmium concentrations ranged from 0.01 to 0.20 mg/kg f.m. The most contaminated blueberries were gathered in Bibiela and Miasteczko Śląskie (Table 2 and Table S3). The average cadmium content in blueberries collected from locations situated more than 15 km away (S2) from the ‘Miasteczko Śląskie’ zinc smelter was 0.01 mg/kg f.m., with a range of cadmium concentrations from 0.01 to 0.02 mg/kg f.m. In five samples, the metal content did not exceed the limit of quantification (LOQ). None of the analyzed blueberries exceeded the maximum permissible concentration of cadmium (Table 2 and Table S3). 

The average lead concentration in the analyzed blueberries was 0.52 mg/kg f.m., which was over five times higher than the maximum permissible level. In the case of fifteen samples, the lead content did not exceed the limit of quantification (LOQ). However, concentrations exceeding the maximum permissible level were found in nine blueberry samples, while in the next one, it was equal to the normative value. The most heavily contaminated berries were collected in the forest near Bibiela, where the lead concentration was 1.44 and 1.19 mg/kg f.m., respectively, exceeding the maximum permissible level by 14.4 times and 11.9 times, respectively. The lead concentration in blueberries collected within the 15 km zone (S1) from the ‘Miasteczko Śląskie’ zinc smelter averaged 0.54 mg/kg f.m., exceeding the maximum permissible level by 5.4 times. In five samples, the lead content was below the limit of quantification. Among the remaining samples, as many as nine exceeded the normative value. The most heavily contaminated blueberries were collected in Bibiela and Miasteczko Śląskie (Table 2 and Table S3). Among the eleven examined samples of blueberries collected from locations situated more than 15 km away (S2) from the zinc smelter, the lead concentration did not exceed the limit of quantification (LOQ) in as many as ten samples. Only in the sample from Lubliniec, the lead concentration was 0.31 mg/kg f.m., exceeding the maximum permissible level by three times (Table 2 and Table S3). 

None of the examined blueberries showed concentrations exceeding the limit of quantification (LOQ) for mercury, arsenic, and nickel (Table 2 and Table S3). 

**Figure 3:**
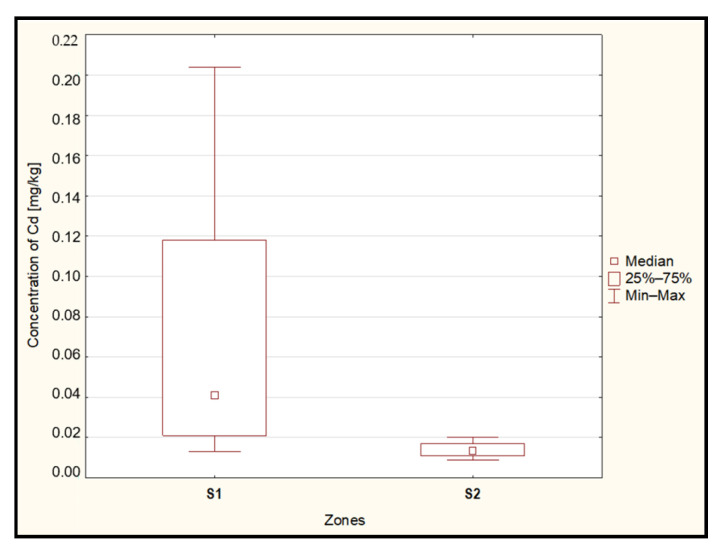
Median concentration of cadmium in the berries from zones located up to 15 km (S1) and above 15 km (S2) from the heavy metal emitter. Source: Own study.

The statistical analysis conducted using the Mann–Whitney U test revealed significantly higher cadmium concentrations (*p* = 0.002) in the examined blueberries originating from the zone located within 15 km (S1) of the ‘Miasteczko Śląskie’ zinc smelter compared to samples collected from the area located more than 15 km away from the source of heavy metal emissions (S2) (Figure 3). 

### 4.2. Health Risk

The assessment of exposure for adult consumers of edible mushrooms from the vicinity of Miasteczko Śląskie and Lubliniec Forests revealed that the average dietary intake of cadmium, lead, and mercury was, respectively, 0.38 µg Cd/kg/day, 0.24 µg Pb/kg/day, and 0.0107 µg Hg/kg/day. In the case of exposure to cadmium and lead, the highest doses were consumed by mushroom consumers from zone 1 (S1), covering an area within a 15 km radius of the ‘Miasteczko Śląskie’ zinc smelter. Only in the case of mercury, greater exposure was observed among mushroom consumers from zone 2 (S2), which includes areas located more than 15 km away from the zinc smelter (Table 3). 

The highest average non-cancer health risk of adult mushroom consumers was estimated concerning exposure to cadmium. The Hazard Quotient (HQ) values ranged from 0.32 to 0.48, depending on the scenario considered. On the other hand, the health risk for consumers ingesting the mushrooms most heavily contaminated with cadmium ranged from HQ = 0.71 to HQ = 1.31. In the case of lead, the health risk varied from 0.32 to 0.42 on average, depending on the analyzed zone. In the scenario assuming the consumption of mushrooms most contaminated with lead, the Hazard Quotient ranged from HQ = 1.08 to HQ = 1.27. On the other hand, the health hazard associated with exposure to mercury was negligible, and even in the scenario considering the consumption of mushrooms most contaminated with mercury, it was HQ = 0.16 (Table 3). The values of uncertainty of Cd, Pb, and Hg ingested doses by adult consumers were in the ranges, respectively, 0.053–0.138, 0.056–0.102, and 0.0035–0.0058. However, uncertainty of HQ in case of Cd, Pb, and Hg were in the ranges 0.053–0.138, 0.896–0.162, and 0.012–0.019. 

The non-cancer health risk assessment for children consuming edible mushrooms from the vicinity of Miasteczko Śląskie and Lubliniec Forests revealed that the highest health hazard was associated with exposure to lead. The average dietary intake for children in terms of cadmium, lead, and mercury was, respectively, 0.89 µg Cd/kg/day, 0.55 µg Pb/kg/day, and 0.0248 µg Hg/kg/day (Table 3). The hazard quotient values indicating significant health risk (HQ >1) were observed exclusively in the case of exposure to the average dose of lead (HQ = 1.10), and in zone S1, both for lead (HQ = 1.23) and cadmium (HQ = 1.12). In the scenario assuming children consume mushrooms most heavily contaminated with cadmium, the HQ value was very high, ranging from 1.65 to 3.04. Meanwhile, the non-neoplastic health risk associated with exposure to the highest doses of lead was even higher, ranging from 3.17 to 3.72. Furthermore, exposure to mercury found in the analyzed mushrooms did not pose a significant health hazard, as the highest estimate was at HQ = 0.37 (Table 3). The uncertainty values of Cd, Pb, and Hg doses ingested by children were in the ranges, respectively, 0.115–0.321, 0.131–0.242, and 0.0083–0.012. The uncertainty of HQ in case of Cd, Pb, and Hg were in the ranges 0.115–0.321, 0.262–0.484, and 0.028–0.041. 

Both the exposure and health risk for adult consumers of blueberries from the vicinity of Miasteczko Śląskie and Lubliniec forests were significantly lower than in scenarios assuming the consumption of edible mushrooms. The average dietary intake of cadmium and lead was 0.002 µg Cd/kg/day and 0.02 µg Pb/kg/day, respectively. In none of the exposure scenarios was a significant health risk demonstrated. Overall, higher exposure and risk were estimated for consumers of blueberries originating from the zone within a radius of 15 km (S1) from the zinc smelter in Miasteczko Śląskie, compared to those consuming blueberries collected from areas farther away from the smelter (S2) (Table 4). The uncertainty values of Cd and Pb ingested doses were in the ranges, respectively, 0.000063–0.00067 and 0.0046–0.0061 in the case of adult consumers, whereas they were in the ranges 0.00015–0.0066 and 0.011–0.014 in the case of children. However, the HQ uncertainty values of Cd and Pb were in the ranges 0.000063–0.00067 and 0.0072–0.0097 in the case of adults, and 0.00015–0.0066 and 0.022–0.029 in the case of children. 

Moreover, children’s exposure to cadmium and lead in the consumed blueberries did not pose a significant health hazard, regardless of the considered exposure scenario. The highest exposure, constituting approximately 27% of the threshold dose, was estimated in the scenario assuming the consumption of blueberries most heavily contaminated with lead (HQ = 0.27). The non-cancer health risk to children resulting from exposure to cadmium ranged from 0.0008 to 0.019, while the health hazard induced by exposure to lead ranged from 0.017 to 0.27 (Table 4). 

**Figure 4:**
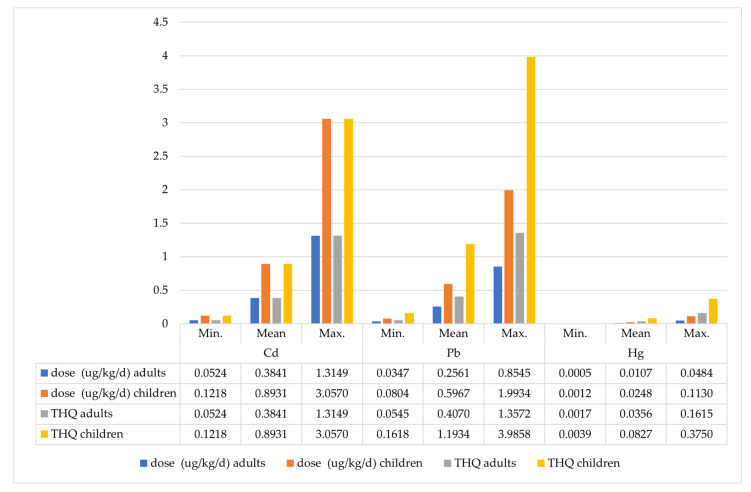
Total exposure and THQ of adults and children to heavy metals in wild mushrooms and blueberries, depending on exposure scenario.

In the next step, adult consumers’ Total Hazard Quotient (THQ) was calculated, representing the sum of exposure to the examined metals from consuming mushrooms and blueberries. Depending on the exposure scenario, the total dose of cadmium ranged from 0.05 to 1.32 µg Cd/kg/day. Significant health risks (THQ > 1) were estimated in scenarios assuming the consumption of mushrooms and blueberries with maximum total cadmium content, as well as in the case of consuming the most heavily contaminated mushrooms and blueberries collected in zone (S1). The total exposure to lead for adults ranged from 0.04 to 0.85 µg Pb/kg/day, translating to risks (THQ) ranging from 0.05 to 1.36. Significant non-cancer health risks associated with lead exposure were observed in consumers of the most heavily contaminated produce from each of the considered zones (S1 and S2). Depending on the exposure scenario, the total mercury dose ranged from 0.0005 to 0.048 µg Hg/kg/day. In none of the scenarios was the hazard quotient higher than THQ > 1, indicating a negligible health hazard. In the scenario assuming adult consumption of the most mercury-contaminated mushrooms and blueberries, THQ = 0.16 (Table 5; Figure 4). 

In the case of children, the total cadmium intake, along with consumed mushrooms and blueberries, varied from 0.12 to 3.06 µg Cd/kg/day, depending on the exposure scenario. Significant non-cancer health risk (THQ = 1.13) was estimated in the scenario considering the average cadmium concentration in blueberries and mushrooms in zone 1 (S1), as well as in all three exposure scenarios involving children’s consumption of forest ground cover produce with maximum cadmium content (THQ from 1.66 to 3.06) (Table S4). 

The dietary intake of lead by children through mushrooms and blueberries ranged from 0.08 to 1.99 µg Pb/kg/day. A substantial non-cancer health hazard for children was found in scenarios involving the consumption of forest ground cover produce with moderate overall lead content and those gathered in zone 1 (S1) (THQ ranging from 1.19 to 1.33).

Consuming produce with moderate lead content from zone 2 (S2) was associated with a health risk very close to the threshold value (THQ = 0.95). A significant non-cancer health risk was also posed by the consumption of mushrooms and blueberries with maximum lead content by children, regardless of the analyzed exposure scenario (THQ from 3.44 to 3.99) (Table S4 and Figure 4).

The total dietary exposure of children to mercury ranged from 0.0008 to 0.113 µg Hg/kg/day. In none of the exposure scenarios was a significant health risk observed. In scenarios considering the maximum mercury content in forest ground cover produce consumed by children, the THQ value ranged from 0.18 to 0.37. The interesting fact is that the consumption of the most contaminated produce from zone 2 (S2) was associated with more than twice the risk compared to consuming mushrooms and blueberries from the area closer to the zinc smelter (Table S4 and Figure 4). 

Additionally, the Hazard Index (HI) was estimated as the cumulative effect of the combined exposure of adults to cadmium, lead, and mercury through the consumption of contaminated edible mushrooms and blueberries. In scenarios assuming minimal and moderate metal content in the examined produce, the highest Hazard Index (HI) value was observed in Zone 1 (S1). Only in the scenario considering maximum metal concentrations was the highest Hazard Index (HI) estimated, assuming overall metal contents in forest ground cover produce (Table 5). 

A significant health risk for adult consumers of forest ground cover produce was estimated in scenarios considering maximum metal concentrations in edible mushrooms and blueberries (HI ranging from 2.16 to 2.83). Results close to the threshold value (HI = 0.86 and 0.96) were calculated in scenarios assuming consumption of overall forest ground cover produce with moderate metal content and in zone 1 (S1) (Table 5). 

The Hazard Index (HI) value for children exposed to metals in consumed forest ground cover produce from the vicinity of Miasteczko Śląskie and Lubliniec forests was significantly higher compared to adults. A significant non-cancer health risk was observed in children consuming both blueberries and mushrooms with moderate and maximum levels of cadmium, lead, and mercury. The Hazard Index (HI) ranged from 1.6 to 2.5 in exposure scenarios considering moderate metal content. The highest risk was observed in children consuming produce gathered within 15 km (S1) from the zinc smelter ‘Miasteczko Śląskie’ S.A. In scenarios considering maximum metal concentrations in forest ground cover produce, the Hazard Index (HI) reached very high values, ranging from 5.8 to 7.4. In this case, the highest health hazard for children was estimated in the scenario assuming the consumption of overall forest ground cover produce, and the lowest in the scenario considering the consumption of produce from zone 2 (S2) (Table 5). 

**Figure 5:**
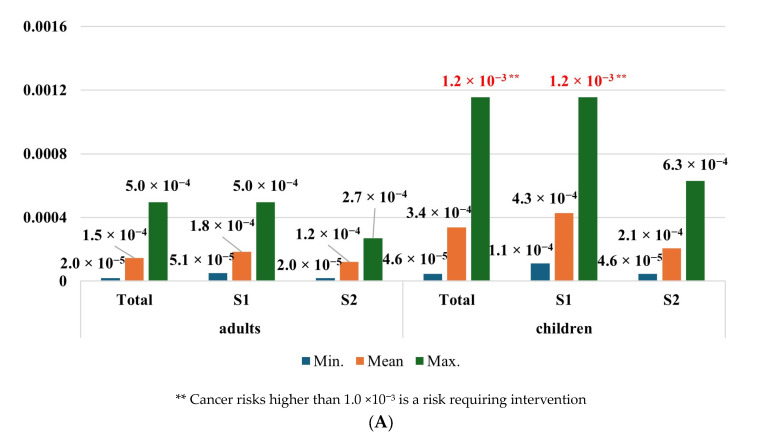

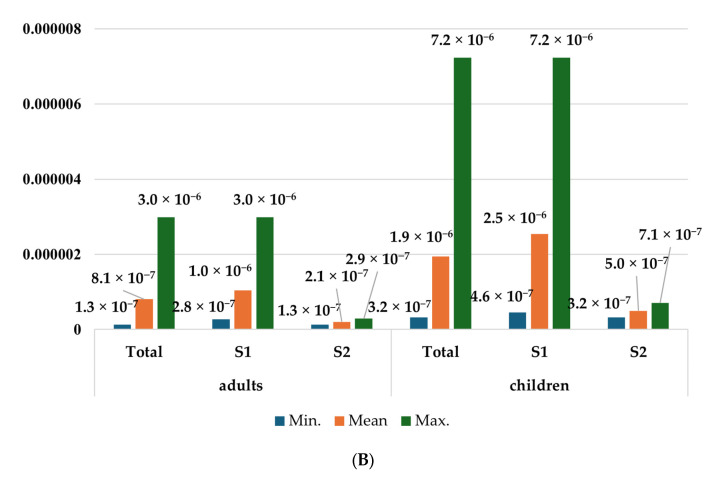
Cancer risk of adults and children from exposure to cadmium in consumed (**A**) wild edible mushrooms and (**B**) blueberries. Source: Own study.

The cancer risk from cadmium ingestion with blueberries in both adults and children was negligible. Depending on the scenario, CR ranged from 1.30 × 10^−7^ to 2.99 × 10^−6^ in the population of adults and from 3.19 × 10^−7^ to 7.23 × 10^−6^ among children. Nevertheless, a significant cancer risk was observed in the case of wild edible mushroom consumption by children. CR values, depending on the scenario, were from 4.59 × 10^−5^ to 1.15 × 10^−3^. The highest cancer risk was observed in children consuming edible mushrooms in the scenario considering maximum metal concentrations from zone 1. The CR values in the population of adults were from 1.97 × 10^−5^ to 4.97 × 10^−4^, which were slightly higher than the acceptable threshold of carcinogenic chemical health risks (Figure 5).

## 5. Discussion

The conducted research indicates that the research hypothesis adopted, assuming the possibility of heavy metal accumulation in forest ground cover produce, i.e., in edible mushrooms and blueberries growing in the forests within the impact range of the zinc smelter in Miasteczko Śląskie and in the Lubliniec Forests, is true. The collected material for testing included samples taken from sites located at a distance of 2.5 km from the smelter, up to samples taken at a distance of 46 km from the emitter. The results indicate that the issue primarily concerned cadmium, and, to a lesser extent, lead. The contamination shown may pose a high health risk to children who regularly consume mushrooms and berries harvested in the studied area. Mushrooms had a greater contribution to dietary exposure to heavy metals compared to blueberries. This results from both the higher cadmium and lead content in mushrooms and the greater mass of consumed mushrooms compared to blueberries. In the case of the analyzed mushrooms, it was found that the average cadmium and lead content in samples collected within a radius of 15 km from the zinc smelter ‘Miasteczko Śląskie’ S.A. was higher compared to the average concentration observed in mushrooms from forests located farther away from the smelter. However, the observed differences were not statistically significant, due to significant variability in the metal concentrations in the analyzed mushrooms, as reflected in high standard deviations. Similar observations were made for the examined berries, with the difference that the cadmium content was significantly higher in samples collected within a 15-km zone surrounding the zinc smelter, compared to blueberries gathered from locations farther away. The difference in the average cadmium concentrations between the two zones was over fivefold. The findings of other researchers are noteworthy in this context. For instance, they revealed a significant accumulation of heavy metals in the bay bolete (*Xerocomus badius*) within the forest complex near the ‘Miasteczko Śląskie’ zinc smelter. However, much like the current study, no variation in pollutant concentrations was observed based on the distance from the emitter [35]. In the studies conducted in the Świerklaniec Forest Division, a statistically significant impact of the distance from the ‘Miasteczko Śląskie’ zinc smelter on decreasing concentrations of heavy metals in blueberries was observed as the distance from the smelter increased. At a distance of over 10 km, a 10-fold lower lead content, 20-fold lower zinc concentrations, and 25-fold lower cadmium content were observed compared to the immediate vicinity of the smelter [36]. However, in subsequent studies in this area, a significant relationship was demonstrated only in relation to the zinc content, and no such correlations were observed for cadmium and lead [37]. As the results of this study indicate, the most contaminated bilberry fruits were recorded at locations in Miasteczko Śląskie or Bibiela, where metal ores were extracted. Therefore, both the current operation of the zinc smelter and the past metal ore mining activities could have had an impact on environmental pollution and the accumulation of heavy metals in the examined berries. In the case of mushrooms, the most contaminated samples, depending on the metal analyzed, originated from Miasteczko Śląskie, as well as from distant locations such as Kalety or Kokotek. This fact confirms the lack of a significant influence of a factor such as the distance from the emitter on the level of contamination of edible mushrooms. The studies conducted in former Czechoslovakia, still in the 20th century, in the vicinity of a lead smelter, showed a statistically significant impact of distance on the extent of metal accumulation in mushrooms. However, these studies were carried out closer to the industrial plant than in the present work, and in the past the emission levels were higher than they are today [38]. 

Climatic factors such as temperature, precipitation, and wind also have a significant impact on the spread of pollutants [39]. As the literature indicates, the heavy metal concentrations in soils changes consistently in the upwind and downwind directions. Permanent wind promotes the transport of heavy metals in the downwind direction [40].

The results of the conducted non-cancer health risk assessment indicate that cadmium and lead are hazardous sources of exposure for consumers of forest ground cover produce from the studied areas. It was demonstrated that higher doses of cadmium and lead enter consumers’ bodies due to consuming mushrooms, compared to berries. It was estimated that in the case of consuming mushrooms with average cadmium and lead content adult consumers ingested doses constituting approximately 32–48% of the safety threshold. However, in exposure scenarios assuming the consumption of the most contaminated mushrooms, the estimated doses of both metals constituted approximately 71–131% of the reference dose of cadmium and approx. 108–170% of the Benchmark Dose Limit (BMDL) for lead. The situation is much more significant from the perspective of children’s health risk. Considering the average cadmium and lead content in the analyzed mushrooms, the absorbed metal dose amounted to a maximum of 112% and 123% of the threshold dose, respectively. However, assuming the consumption of the most contaminated mushrooms by children, the calculated health risk ranged from 165% to 304% for cadmium and from 317% to 372% for lead. According to the research results of Gałgowska and Pietrzak-Fiećko (2021), the exposure of adults consuming edible mushrooms from the Warmian–Masurian Voivodeship accounted for 35.4% of the Tolerable Weekly Intake of cadmium (2.5 µg/kg b.m./week) and 0.63% of the Provisional Tolerable Weekly Intake of lead (25 µg/kg b.m./week) under the conditions of consuming the most contaminated mushrooms [41]. The exposure of mushroom consumers in the Warmian–Masurian Voivodeship to lead is negligible, while consuming the most contaminated mushrooms from the Silesian Voivodeship may pose a significant health risk. As it turns out, dried mushrooms available on the Polish market, such as boletus or porcini mushrooms, can also be a source of exposure to toxic metals for consumers. The study by Orywal et al. (2021) showed that the average concentrations of Hg, Cd, Pb, and As in Boletus edulis were 3.04 mg Hg/kg, 1.98 mg Cd/kg, 1.16 mg Pb/kg, and 0.90 mg As/kg, respectively. In the case of Xerocomus badius, the concentrations were 0.10 mg Hg/kg, 1.15 mg Cd/kg, 0.93 mg Pb/kg, and 0.28 mg As/kg [42]. 

The assessment of exposure and non-cancer health risk arising from the consumption of berries did not indicate a significant health hazard. In all the assumed exposure scenarios, it was found that the consumption of blueberries by both adults and children did not result in the intake of doses that would be hazardous to health It is only worth paying attention to children consuming the most contaminated berries from Bibela and Miasteczko Śląskie, who potentially received doses of lead amounting to approx. 27% of the threshold dose. This result should be considered dangerous, because the included mass of blueberries consumed by children in the exposure assessment was relatively small. Additionally, berries only serve as a supplement to the diet and are not its primary component. As the literature indicates, the concentration of metals in blueberry fruits and mushrooms [43] reflects the degree of soil contamination. Therefore, it can be assumed that there is a very high content of toxic metals in the forest ground cover substrate of the study areas. This is confirmed by previous studies conducted by Pająk and Jasik (2010) in the neighborhood of the zinc smelter ‘Miasteczko Śląskie’ S.A. and Piekut et al. (2018) in Tarnowskie Gory County (Miasteczko Slaskie). High concentrations of heavy metals were found in both forest soil (average concentrations: 117.43 mg Zn/kg; 2.93 mg Cd/kg; 176.64 mg Pb/kg) [37] and soil samples from arable fields (average concentrations of Cd: 9.25 mg Cd/kg) [44]. In the study by Nitu et al. (2022), it was found that the accumulation of metals in blueberries can be ranked in the following order: Zn > Cu > Pb [45]. Thus, with the low mobility of lead in the environment, a sufficiently high metal concentration was observed in the examined berries, translating into a significant exposure dose for children.

The conclusion of the conducted health risk assessment was based on the estimates of the Total Hazard Quotient (THQ) and the Hazard Index (HI). The value of THQ allows us to assess the magnitude of health risk associated with exposure to a single metal present in both consumed mushrooms and blueberries. On the other hand, HI is an indicator relating to the magnitude of the health hazard due to exposure to all the examined metals combined, as a result of consuming both edible mushrooms and berries collected in the studied forest area. Based on the conducted research, it can be concluded that severe health risks apply to adult consumers due to exposure to cadmium in the most contaminated forest ground cover produce collected in zone S1, as well as due to exposure to lead in the most contaminated mushrooms and berries from both zones. In the case of children, a THQ value ≥ 1, indicating a significant non-cancer health risk, was demonstrated in connection with the consumption of the most contaminated forest ground cover produce with cadmium and lead from both zones. This risk was also evident in scenarios assuming the consumption of produce with moderate cadmium content collected from zone S1. On the other hand, considering exposure to the full spectrum of the examined metals, significant health risks were found in the group of adult consumers of forest ground cover produce who consumed the studied mushrooms and berries with the highest contamination, regardless of the sampling location. Moreover, in the case of consuming samples from zone S1, the assumed metal doses were HI = 0.96, indicating that they were very close to the threshold dose representing a health-threatening level of exposure to metals. For children consuming the forest ground cover produce from the studied areas, significant health risks result from consuming both the produce with moderate metal content from both zones and mushrooms and berries with the maximum metal content. The values of the HI index for scenarios assuming the consumption of the produce with moderate concentrations ranged from 1.6 to 2.5, and in scenarios with maximum concentrations it ranged from 5.8 to 7.4, indicating a very high and alarming health risk. 

The conducted research highlights the necessity to refrain from consuming, particularly, edible mushrooms growing in the vicinity of Miasteczko Śląskie and in the forests of Lubliniec. Regular consumption of these may be associated with a sufficiently high dietary exposure to toxic metals, leading to an increased likelihood of developing adverse health effects. Children should absolutely avoid consuming mushrooms from the studied area, as they may experience exposure translating into very high health risks. Consuming berries from the studied forests poses a lesser health hazard, although in some cases blueberries may deliver high metal doses to the body, especially in children. In this case, it has been demonstrated that the distance from the heavy metal emitter, namely the zinc smelter, is significant. In light of the above, the limitation of consumption should also apply to blueberries collected in forests within a radius of 15 km from Miasteczko Śląskie, while consuming berries from more distant forests should be limited to a minimum. One way to at least partially reduce the exposure of forest ground cover fruit consumers to heavy metals is to wash the fruit thoroughly before consumption. Additionally, it is crucial to raise awareness among the residents of Miasteczko Śląskie and the neighboring towns adjacent to the studied forest complexes regarding the existing risk associated with exposure to heavy metals and ways to mitigate health risks. 

## 6. Conclusions and Future Outlook

- The conducted research revealed high concentrations of cadmium and lead in the examined edible mushrooms and berries from the forests in Miasteczko Śląskie and the Lubliniec region. However, the content of mercury, arsenic, and nickel in the tested samples was negligible;
- A statistically significant impact of the distance from ‘Miasteczko Śląskie’ S.A. zinc smelter on the variation in cadmium concentration in the examined berries was observed;
- The exposure of adult consumers to the most contaminated mushrooms with cadmium and lead poses a significant non-cancer health risk. A much higher health risk was noted in the case of children, among whom a significant health risk was identified in the scenario considering the consumption of mushrooms with moderate cadmium and lead content;
- The level of exposure of adult consumers and children to the studied metals in the consumed berries did not pose a significant health hazard;
- The overall non-cancer health risk arising from the combined exposure of adults to all the investigated metals found in consumed mushrooms and berries was significant when consuming the most heavily contaminated produce. In contrast, among children, the risk was significant even when consuming the forest ground cover produce with average levels of the analyzed metals;
- Dietary exposure of children to cadmium consuming the most contaminated wild mushrooms was associated with a high cancer health risk;
- In order to reduce the health risk to children from the forests in Miasteczko Śląskie and the Lubliniec region, parents should follow the recommendations regarding the ban on wild mushroom consumption by children, especially mushrooms from local sources;
- It is recommended to take action to increase awareness among residents of the areas adjacent to the studied forests regarding the existing health hazard and possible measures to minimize exposure to metals present in mushrooms and berries from local forest ecosystems;
- Further studies are needed to investigate the correlation between heavy metal concentrations in mushrooms/forest fruits and in the soil, taking into consideration wind conditions and different distances from the smelter.

## Figures and Tables

**Table 1:** The values of RfD and BMDL used in the non-cancer health risk assessment.

Metal	The Threshold Dose	[µg/kg per Day]	References
**Cd**	RfD	1.00	[29]
**Pb**	BMDL_10_ adults	0.63	[30]
	BMDL_01_ children	0.50	[30]
**As**	RfD	0.30	[31]
**Ni**	RfD	0.02	[32]
**Hg**	RfD	0.30	[33]

**Table 2:** Heavy metals concentrations in the samples of the edible fungi and berries.

	Edible Fungi					Berries				
	[mg/kg/Fresh Mass]					[mg/kg/Fresh Mass]				
	Cd	Pb	Hg	As	Ni	Cd	Pb	Hg	As	Ni
Mean ± SD Range (min–max)	0.98 ± 0.82 0.13–3.35	0.60 ± 0.58 0.08–2.05	0.0274 ± 0.0407 0.0013–0.1242	ND ND	ND ND	0.05 ± 0.06 0.01–0.20	0.52 ± 0.48 0.09–1.44	ND ND	ND ND	ND ND
Maximum Allowable Concentration [34]	0.50	0.80	0.50	0.50	ND	0.03	0.10	0.01	ND	ND

ND—no data.

**Table 3:** Dietary exposure of adults and children to heavy metals from mushrooms depending on the location of the samples.

	Exposure Scenario [µg/kg/Day]				HQ			
Metal	Scenario	Total	S1	S2	Scenario	Total	S1	S2
**ADULTS**								
**Cd**	Min.	0.05	0.14	0.05	Min.	0.05	0.14	0.05
	Mean	0.38	0.48	0.32	Mean	0.38	0.48	0.32
	Max.	1.31	1.31	0.71	Max.	1.31	1.31	0.71
**Pb**	Min.	0.03	0.03	0.04	Min.	0.05	0.05	0.06
	Mean	0.24	0.26	0.20	Mean	0.38	0.42	0.32
	Max.	0.80	0.68	0.80	Max.	1.27	1.08	1.27
**Hg**	Min.	0.0005	0.0006	0.0005	Min.	0.0017	0.0019	0.0017
	Mean	0.0107	0.0053	0.0139	Mean	0.0356	0.0177	0.0463
	Max.	0.0484	0.0231	0.0484	Max.	0.1615	0.0769	0.1615
**CHILDREN**								
**Cd**	Min.	0.12	0.30	0.12	Min.	0.12	0.30	0.12
	Mean	0.89	1.12	0.54	Mean	0.89	1.12	0.54
	Max.	3.04	3.04	1.66	Max.	3.04	3.04	1.66
**Pb**	Min.	0.07	0.07	0.09	Min.	0.15	0.15	0.18
	Mean	0.55	0.61	0.45	Mean	1.10	1.23	0.89
	Max.	1.86	1.59	1.86	Max.	3.72	3.17	3.72
**Hg**	Min.	0.0012	0.0014	0.0008	Min.	0.0039	0.0045	0.0026
	Mean	0.0248	0.0124	0.0259	Mean	0.0827	0.0412	0.0866
	Max.	0.1130	0.0537	0.1130	Max.	0.3750	0.179	0.3750

S1—Scenario Zone 1; S2—Scenario Zone 2; HQ—Hazard Quotient.

**Table 4:** Dietary exposure of adult and children to heavy metals from berries depending on the location of the samples.

	Exposure Scenario [µg/kg/day]				HQ			
Metal	Scenario	Total	S1	S2	Scenario	Total	S1	S2
**ADULTS**								
**Cd**	Min.	0.0004	0.0007	0.0004	Min.	0.0004	0.0007	0.0004
	Mean	0.0021	0.0028	0.0005	Mean	0.0021	0.0028	0.0005
	Max.	0.0079	0.0079	0.0008	Max.	0.0079	0.0079	0.0008
**Pb**	Min.	0.0035	0.0035	0.0120	Min.	0.0055	0.0055	0.0190
	Mean	0.0201	0.0209	0.0120	Mean	0.0320	0.0332	0.0190
	Max.	0.0555	0.0555	0.0120	Max.	0.0882	0.0882	0.0190
**CHILDREN**								
**Cd**	Min.	0.0008	0.0012	0.0008	Min.	0.0008	0.0012	0.0008
	Mean	0.0051	0.0067	0.0013	Mean	0.0051	0.0067	0.0013
	Max.	0.0190	0.0190	0.0019	Max.	0.0190	0.0190	0.0019
**Pb**	Min.	0.0084	0.0084	0.0289	Min.	0.0168	0.0168	0.0579
	Mean	0.0487	0.0507	0.0289	Mean	0.0974	0.1014	0.0579
	Max.	0.1344	0.1344	0.0289	Max.	0.2688	0.2688	0.0579

S1—Scenario Zone 1; S2—Scenario Zone 2; HQ—Hazard Quotient.

**Table 5:** Hazard Index of children and adult consumers of mushrooms and berries contaminated by heavy metals, depending on the location of the samples.

Population	Exposure Scenario	HI		
		Total	S1	S2
*Adults*	Min.	0.11	0.19	0.13
	Mean	0.83	0.96	0.70
	Max.	2.83	2.56	2.16
*Children*	Min.	0.29	0.46	0.36
	Mean	2.17	2.50	1.58
	Max.	7.42	6.68	5.81

HI—Hazard Index.

## Data Availability

The data that support the findings of this study are available from the corresponding author upon responsible request.

## Supplementary Materials

The following supporting information can be downloaded at: https://www.mdpi.com/article/10.3390/toxics12020101/s1. Table S1: The distance of the edible fungi and berries sample collection station from the Zinc Smelter ‘Miasteczko Śląskie’ S.A. Table S2: Heavy metals content in the samples of the edible fungi. Table S3: Heavy metals content in the samples of the berries. Table S4: Total exposure and THQ of adult and children to heavy metals on mushrooms and berries depending on the location of the samples.
