# Supplementary material for: Health Risks for Consumers of Forest Ground Cover Produce Contaminated with Heavy Metals
Source: Toxics. 2024 Jan 24;12(2):101. doi: 10.3390/toxics12020101 (PMC10892603; doi:10.3390/toxics12020101)
Supplement: Supplementary file 1 [file toxics-12-00101-s001.zip › toxics-2778125-supplementary.pdf]

Supplementary Materials

Table S1. The distance of the edible fungi and berries sample collection station from the Zinc Smelter 'Miasteczko Śląskie' S.A.

Sample no.		Material	Location	Distance from zinc smelter (km)
1.	Mushrooms	Podgrzybek zajączek	50°28'07.3"N 18°55'15.2"E	6
2.		Podgrzybek brunatny	50°38'52.5"N 18°41'19.9"E	37
3.		Podgrzybek zajączek	50°39'57.6"N 18°38'05.4"E	40
4.		Podgrzybek brunatny	50°37'31.8"N 19°03'39.3"E	23
5.		Kozak czerwony	50°37'44.4"N 18°40'54.7"E	35
6.		Borowik szlachetny	50°34'36.5"N 18°52'01.5"E	13
7.		Maślak	50°34'40.2"N 18°51'54.4"E	15
8.		Maślak	50°35'50.7"N 18°42'27.5"E	31
9.		Borowik szlachetny	50°35'53.0"N 18°42'29.5"E	31
10.		Maślak	50°29'18.8"N 18°54'29.8"E	3
11.		Podgrzybek brunatny	50°29'18.7"N 18°54'30.0"E	3
12.		Podgrzybek zajączek	50°39'53.7"N 18°37'51.7"E	39
13.		Maślak	50°34'37.5"N 18°51'58.7"E	14
14.		Podgrzybek zajączek	50°42'36.8"N 18°40'49.0"E	39
15.		Borowik szlachetny	50°45'26.1"N 18°36'24.1"E	46
16.		Podgrzybek zajączek	50°33'07.7"N 18°57'15.6"E	8
17.		Podgrzybek zajączek	50°37'42.4"N 18°41'03.0"E	35
18.		Kozak siwy	50°35'54.8"N 18°42'38.4"E	31
19.		Podgrzybek brunatny	50°39'39.9"N 18°40'41.4"E	36
20.		Podgrzybek brunatny	50°35'49.0"N 18°42'25.0"E	31
21.		Podgrzybek zajączek	50°29'18.9"N 18°54'32.8"E	2.50
22.		Maślak	50°29'47.3"N 18°55'39.5"E	0.65
23.		Kozak siwy	50°45'31.7"N 18°36'18.2"E	46
24.		Maślak	50°29'17.9"N 18°54'29.6"E	3
25.		Podgrzybek brunatny	50°39'11.8"N 18°41'25.2"E	37
26.		Podgrzybek zajączek	50°35'12.0"N 18°48'51.5"E	23
1.		Berries	50°29'19.2"N 18°54'32.5"E	2.50
2.			50°39'57.5"N 18°38'02.1"E	40
3.			50°39'12.5"N 18°34'48.4"E	43
4.			50°39'16.6"N 18°42'14.6"E	39
5.			50°29'19.2"N 18°54'37.2"E	3
6.			50°39'10.5"N 18°34'55.6"E	39
7.			50°39'10.5"N 18°34'55.6"E	44
8.			50°39'42.0"N 18°39'16.1"E	0.40
9.			50°29'19.0"N 18°54'34.0"E	2.50
10.			50°34'37.5"N 18°51'58.7"E	14
11.			50°29'18.9"N 18°54'35.4"E	3
12.			50°35'38.5"N 18°58'20.9"E	13
13.			50°28'06.4"N 18°55'13.3"E	4.50
14.			50°34'53.8"N 19°02'40.6"E	17
15.			50°37'31.4"N 19°04'38.8"E	23
16.			50°29'19.3"N 18°54'33.6"E	2.50
17.			50°38'53.5"N 18°43'07.3"E	35
18.			50°35'38.8"N 18°58'22.8"E	13
19.			50°39'15.1"N 18°42'18.8"E	36
20.			50°34'34.3"N 18°52'02.9"E	0.30
21.			50°39'10.3"N 18°42'32.3"E	34
22.			50°34'38.1"N 18°51'57.1"E	14
23.			50°35'37.4"N 18°58'18.8"E	12
24.			50°35'38.6"N 18°58'24.3"E	13
25.			50°30'07.3"N 19°00'27.9"E	8
26.			50°30'06.3"N 19°00'29.4"E	9

Source: Own study.

Table S2. Heavy metals content in the samples of the edible fungi.

Sample no.	Location	Distance from smelter (km)	Metal concentration [mg/kg fresh mass]				
			Cd	Pb	Hg	As	Ni
1	50°28'07.3"N 18°55'15.2"E	6	1.52	0.34	0.0056	<0.43	<0.83
2	50°38'52.5"N 18°41'19.9"E	37	0.80	<0.08	<0.0005	<0.43	<0.83
3	50°39'57.6"N 18°38'05.4"E	40	0.47	<0.08	<0.0005	<0.43	<0.83
4	50°37'31.8"N 19°03'39.3"E	23	1.41	0.53	0.0824	<0.43	<0.83
5	50°37'44.4"N 18°40'54.7"E	35	0.45	0.14	0.0218	<0.43	<0.83
6	50°34'36.5"N 18°52'01.5"E	13	3.35	0.58	0.0592	<0.43	<0.83
7	50°34'40.2"N 18°51'54.4"E	15	0.88	1.75	<0.0005	<0.43	<0.83
8	50°35'50.7"N 18°42'27.5"E	31	0.13	2.05	0.0013	<0.43	<0.83
9	50°35'53.0"N 18°42'29.5"E	31	1.83	0.37	0.1242	<0.43	<0.83
10	50°29'18.8"N 18°54'29.8"E	3	0.33	0.93	0.0066	<0.43	<0.83
11	50°29'18.7"N 18°54'30.0"E	3	0.70	0.65	<0.0005	<0.43	<0.83
12	50°39'53.7"N 18°37'51.7"E	39	1.04	<0.08	0.0069	<0.43	<0.83
13	50°34'37.5"N 18°51'58.7"E	14	0.68	0.08	0.0016	<0.43	<0.83
14	50°42'36.8"N 18°40'49.0"E	39	0.14	<0.08	0.0020	<0.43	<0.83
15	50°45'26.1"N 18°36'24.1"E	46	0.72	0.23	0.1012	<0.43	<0.83
16	50°33'07.7"N 18°57'15.6"E	8	1.53	<0.08	0.0015	<0.43	<0.83
17	50°37'42.4"N 18°41'03.0"E	35	0.31	0.10	0.0020	<0.43	<0.83
18	50°35'54.8"N 18°42'38.4"E	31	0.68	<0.08	0.0022	<0.43	<0.83
19	50°39'39.9"N 18°40'41.4"E	36	0.22	0.15	<0.0005	<0.43	<0.83
20	50°35'49.0"N 18°42'25.0"E	31	1.38	<0.08	<0.0005	<0.43	<0.83
21	50°29'18.9"N 18°54'32.8"E	2.5	3.01	0.14	<0.0005	<0.43	<0.83
22	50°29'47.3"N 18°55'39.5"E	0.65	0.35	0.84	<0.0005	<0.43	<0.83
23	50°45'31.7"N 18°36'18.2"E	46	1.61	<0.08	0.0122	<0.43	<0.83
24	50°29'17.9"N 18°54'29.6"E	3	0.38	0.79	0.0073	<0.43	<0.83
25	50°39'11.8"N 18°41'25.2"E	37	1.33	<0.08	<0.0005	<0.43	<0.83
26	50°35'12.0"N 18°48'51.5"E	23	0.58	<0.08	<0.0005	<0.43	<0.83
	MEAN		0.98	0.60	0.0274	<0.43	<0.83
	SD		0.82	0.58	0.0407	ND	ND
	MINIMUM CONCENTRATION		0.13	0.08	0.0013	ND	ND
	MAXIMUM CONCENTRATION		3.35	2.05	0.1242	ND	ND
	MAXIMUM ALLOWABLE CONCENTRATION [34]*		0.50*	0.80*	0.50*	0.50*	ND

SD – standard deviation
ND – no data
Source: Own study.

Table S3. Heavy metals content in the samples of the berries.

Sample No.	Localization	Distance from smelter (km)	Metal concentration [mg/kg fresh mass]				
			Cd	Pb	Hg	As	Ni
1	50°29'19.2"N 18°54'32.5"E	2.5	0.067	0.58	<0.0005	<0.43	<0.83
2	50°39'57.5"N 18°38'02.1"E	40	0.020	<0.08	<0.0005	<0.43	<0.83
3	50°39'12.5"N 18°34'48.4"E	43	0.009	<0.08	<0.0005	<0.43	<0.83
4	50°39'16.6"N 18°42'14.6"E	39	0.013	<0.08	<0.0005	<0.43	<0.83
5	50°29'19.2"N 18°54'37.2"E	3	0.118	0.36	<0.0005	<0.43	<0.83
6	50°39'10.5"N 18°34'55.6"E	39	0.014	<0.08	<0.0005	<0.43	<0.83
7	50°39'10.5"N 18°34'55.6"E	44	0.011	<0.08	<0.0005	<0.43	<0.83
8	50°39'42.0"N 18°39'16.1"E	40	0.017	<0.08	<0.0005	<0.43	<0.83
9	50°29'19.0"N 18°54'34.0"E	2.5	0.045	0.22	<0.0005	<0.43	<0.83
10	50°34'37.5"N 18°51'58.7"E	14	0.034	<0.08	<0.0005	<0.43	<0.83
11	50°29'18.9"N 18°54'35.4"E	3	0.184	1.06	<0.0005	<0.43	<0.83
12	50°35'38.5"N 18°58'20.9"E	13	0.020	<0.08	<0.0005	<0.43	<0.83
13	50°28'06.4"N 18°55'13.3"E	4.5	0.033	0.10	<0.0005	<0.43	<0.83
14	50°34'53.8"N 19°02'40.6"E	17	<0.008	<0.08	<0.0005	<0.43	<0.83
15	50°37'31.4"N 19°04'38.8"E	23	<0.008	<0.08	<0.0005	<0.43	<0.83
16	50°29'19.3"N 18°54'33.6"E	2.5	0.043	0.21	<0.0005	<0.43	<0.83
17	50°38'53.5"N 18°43'07.3"E	35	<0.008	0.31	<0.0005	<0.43	<0.83
18	50°35'38.8"N 18°58'22.8"E	13	0.013	<0.08	<0.0005	<0.43	<0.83
19	50°39'15.1"N 18°42'18.8"E	36	<0.008	<0.08	<0.0005	<0.43	<0.83
20	50°34'34.3"N 18°52'02.9"E	13	0.038	0.18	<0.0005	<0.43	<0.83
21	50°39'10.3"N 18°42'32.3"E	34	<0.008	<0.08	<0.0005	<0.43	<0.83
22	50°34'38.1"N 18°51'57.1"E	14	0.041	0.09	<0.0005	<0.43	<0.83
23	50°35'37.4"N 18°58'18.8"E	12	0.019	<0.08	<0.0005	<0.43	<0.83
24	50°35'38.6"N 18°58'24.3"E	13	0.021	<0.08	<0.0005	<0.43	<0.83

25	50°30'07.3"N 19°00'27.9"E	8	0.204	1.44	<0.0005	<0.43	<0.83
26	50°30'06.3"N 19°00'29.4"E	9	0.190	1.19	<0.0005	<0.43	<0.83
MEAN			0.05	0.52	ND	ND	ND
SD			0.06	0.48	ND	ND	ND
MINIMUM CONCENTRATION			0.01	0.09	ND	ND	ND
MAXIMUM CONCENTRATION			0.20	1.44	ND	ND	ND
MAXIMUM ALLOWABLE CONCENTRATION [34]*			0.03*	0.10*	0.01*	ND	ND

SD – standard deviation
ND – no data
Source: Own study.

Table S4. Total exposure and THQ of adult and children to heavy metals on mushrooms and berries depending on the location of the samples.

**Metal**	**Scenario**	**Exposure Scenario [µg/kg/day]**			**THQ**			
		**Total**	**S1**	**S2**	**Scenario**	**Total**	**S1**	**S2**
				Adults				
	Min.	0.052	0.136	0.052	Min.	0.05	0.14	0.05
Cd	Mean	0.384	0.486	0.318	Mean	0.38	0.49	0.32
	Max.	1.315	1.315	0.712	Max.	1.32	1.32	0.71
	Min.	0.035	0.035	0.051	Min.	0.05	0.05	0.08
Pb	Mean	0.256	0.285	0.211	Mean	0.41	0.45	0.34
	Max.	0.854	0.737	0.811	Max.	1.36	1.17	1.29
	Min.	0.0005	0.0006	0.0005	Min.	0.0017	0.0019	0.0017
Hg	Mean	0.0107	0.0053	0.0139	Mean	0.0356	0.0177	0.0463
	Max.	0.0484	0.0231	0.0484	Max.	0.1615	0.0769	0.1615
				Children				
	Min.	0.12	0.30	0.12	Min.	0.12	0.30	0.12
Cd	Mean	0.89	1.13	0.55	Mean	0.89	1.13	0.55
	Max.	3.06	3.06	1.66	Max.	3.06	3.06	1.66
	Min.	0.08	0.08	0.12	Min.	0.16	0.16	0.24
Pb	Mean	0.60	0.67	0.48	Mean	1.19	1.33	0.95
	Max.	1.99	1.72	1.89	Max.	3.99	3.44	3.78
	Min.	0.0012	0.0014	0.0008	Min.	0.0039	0.0045	0.0026
Hg	Mean	0.0248	0.0124	0.0259	Mean	0.0827	0.0412	0.0866
	Max.	0.1130	0.0537	0.1130	Max.	0.3750	0.1790	0.3750

S1 – Scenario Zone 1
S2 – Scenario Zone 2
THQ – Total Hazard Quotient
